# Favourable metabolic profile sustains mitophagy and prevents metabolic abnormalities in metabolically healthy obese individuals

**DOI:** 10.1186/s13098-017-0298-x

**Published:** 2017-12-12

**Authors:** Shipra Bhansali, Anil Bhansali, Veena Dhawan

**Affiliations:** 1 Department of Experimental Medicine and Biotechnology, Postgraduate Institute of Medical Education and Research (PGIMER), Research Block-B, Chandigarh, 160012 India; 2 Department of Endocrinology, Postgraduate Institute of Medical Education and Research (PGIMER), Chandigarh, India

**Keywords:** Metabolically healthy non-obese (MHNO), Metabolically healthy obese (MHO), Metabolically abnormal diabetic obese (MADO), Mitochondrial oxidative stress and mitophagy

## Abstract

**Background:**

Obesity-mediated oxidative stress results in mitochondrial dysfunction, which has been implicated in the pathogenesis of metabolic syndrome and T2DM. Recently, mitophagy, a cell-reparative process has emerged as a key facet in maintaining the mitochondrial health, which may contribute to contain the metabolic abnormalities in obese individuals. However, the status of mitophagy in metabolically healthy obese (MHO) and metabolically abnormal diabetic obese (MADO) subjects remains to be elucidated. Hence, the present study aims to unravel the alterations in mitochondrial oxidative stress (MOS) and mitophagy in these subjects.

**Methods:**

60 subjects including MHNO (metabolically healthy non-obese), MHO and MADO were enrolled as per the Asian criteria for obesity (n = 20 each). Biochemical parameters, MOS indices, transcriptional and translational expression of mitophagy markers (*PINK1*, *PARKIN*, *MFN2*, *NIX*, *LC3*-*II*, and *LAMP*-*2*), and transmission electron microscopic (TEM) studies were performed in peripheral blood mononuclear cells.

**Results:**

The MHO subjects displayed a favorable metabolic profile, despite accompanied by an increased adiposity as compared to the MHNO group; while MADO group exhibited several metabolic abnormalities, inspite of similar body composition as MHO subjects. A progressive rise in the MOS was observed in MHO and MADO subjects as compared to the MHNO group, and it showed a positive and significant correlation with the body composition in these groups. Further, mitophagy remained unaltered in the MHO group, while it was significantly downregulated in the MADO group. In addition, TEM studies revealed a significant increase in the percentage of damaged mitochondria in MADO patients as compared to other groups, while MHO and MHNO groups did not show any significant alterations for the same.

**Conclusion:**

A favorable metabolic profile and moderate levels of MOS in the MHO group may play a crucial role in the sustenance of mitophagy, which may further limit the aggravation of MOS, inflammation, and emergence of metabolic aberrations in contrast to MADO subjects, who exhibited multiple metabolic abnormalities and attenuated mitophagy. Therefore, these MHO subjects are likely to be at a lower risk of developing metabolic syndrome and T2DM.

## Background

Obesity is associated with numerous metabolic alterations such as insulin resistance, glucose intolerance, and dyslipidemia. The constellation of these risk factors constitute the metabolic syndrome that predisposes the individuals to the risk of developing type 2 diabetes mellitus (T2DM) and cardiovascular diseases [1, 2]. However, a subset of obese subjects do not exhibit an increased risk of developing these aforementioned metabolic abnormalities, and they have been identified as metabolically healthy obese (MHO) subjects, accounting for approximately 20–30% of the obese adult population [3]. A study by Zheng et al. reported that the prevalence of MHO subjects was 27.9% in the obese adult Chinese population [4]. Similarly, another study from Southern India described the prevalence of MHO as 13.3% in the adult population [5].

MHO subjects are characterized by the absence of several metabolic abnormalities such as insulin resistance, dyslipidemia, glucose intolerance, hypertension, inspite of increased adiposity, but have a favorable inflammatory profile as compared to the metabolically abnormal diabetic obese (MADO) individuals, who are usually accompanied by severe metabolic abnormalities. However, the underlying mechanisms contributing towards the relative protection against obesity-induced metabolic complications remains largely unknown.

Metabolic syndrome is associated with oxidative stress and mitochondrial dysfunction. Mitochondrion, a vital cell organelle, is the primary site for endogenous ROS generation and glucose metabolism. High calorific diet intake leads to an energetic overload, which in turn results in an ineffective mitochondrial oxidative phosphorylation with an abnormal generation of mitochondrially-derived superoxide anions, eventually damaging the mitochondrial-related DNA and proteins [6]. Moreover, mitochondria are also involved in the insulin biosynthesis and secretion as well as in fuel metabolism at insulin target sites [7]. Recently, a study by Chattopadhyay et al. reported that the mitochondrial reactive oxygen species (ROS) production was higher in the subcutaneous adipose tissue of the obese T2DM subjects as compared to the obese non-diabetic subjects and non-obese T2DM patients, indicating that the mitochondrial dysfunction with the enhanced ROS production may contribute to metabolic abnormalities in obesity and T2DM [8]. Similarly, another study by Befroy et al. reported that the mitochondrial dysfunction is associated with insulin resistance in skeletal muscle of T2DM patients [9].

In order to maintain the mitochondrial health, a process known as mitophagy, and its implications in metabolic syndrome have emerged as a novel area of research. Mitophagy, also known as mitochondrial autophagy involves the selective sequestration of damaged or defunct mitochondria via autophagic vesicles, followed by their subsequent lysosomal degradation, thereby playing a crucial role in the regulation of mitochondrial quality and quantity control [10]. In this context, Twig et al. reported that mitophagy is important in the turnover of dysfunctional mitochondria in β-cells, which is required for its optimal functioning, and the dysregulation of this process might predispose to the risk of developing metabolic syndrome and T2DM [11].

Mitophagy is a multi-step, evolutionary conserved process, regulated by various genes including PTEN-induced putative kinase 1 (*PINK1*), *PARKIN*, NIP3-like protein X (*NIX*) and mitofusin2 (*MFN2*), involved in the priming of the damaged/defunct mitochondria, followed by their recruitment to the autophagosome machinery via another protein, microtubule-associated protein light chain 3 (*LC3*), a marker of autophagosomal membrane. The autophagic vesicles carrying the defunct mitochondria, known as mitophagosomes, subsequently fuse with the lysosomes, which is facilitated by lysosome-associated membrane protein-2 (*LAMP*-*2*) [12]. Thereafter, the engulfed mitochondrial cargo is degraded by lysosomal hydrolases, hence maintaining the mitochondrial health.

Mounting evidence reveal that the obesity and/or metabolic abnormalities-mediated inflammation, and MOS are closely associated with mitochondrial dysfunction [13, 14]. Therefore, the regulated elimination of dysfunctional mitochondria is of primary importance to suppress the obesity-induced metabolic disorders. Based on the above evidence, we hypothesized that mitophagy, a cell-reparative phenomenon, which is essential for restoring the mitochondrial function, might attribute to the pathophysiology of obesity. Hence, the present study aimed to assess the magnitude of mitochondrial oxidative stress and mitophagy in metabolically healthy non-obese (MHNO), metabolically healthy obese (MHO) and metabolically abnormal diabetic obese (MADO) subjects, who were accompanied with varying degree of adiposity and/or metabolic abnormalities.

## Methods

### Study population

Sixty, age, and gender-matched non-obese (BMI < 23 kg/m^2^) and obese (BMI ≥ 25 kg/m^2^) subjects were screened as per the Asian criteria for obesity and enrolled at endocrine out-patient department of the Postgraduate Institute of Medical Education and Research (PGIMER), Chandigarh, India [15]. The revised National Cholesterol Education Programs’s Adult treatment Panel III report for Asian population defined the criteria for the metabolic syndrome as follows: WC > 80 cm in men, > 90 cm in women, BP > 130/85 mmHg, FPG ≥ 100 mg/dl or HbA_1C_ > 5.6%, TG ≥ 150 mg/dl and HDL-C < 40 mg/dl in men or < 50 mg/dl in women [16]. According to the above mentioned criteria, the study participants were divided into 3 groups: (a) Metabolically healthy non-obese (MHNO): BMI < 23 kg/m^2^ and ≤ 1 metabolic risk factor. (b) Metabolically healthy obese (MHO): BMI ≥ 25 kg/m^2^ and ≤ 1 metabolic risk factor. (c) Metabolically abnormal diabetic obese (MADO): BMI ≥ 25 kg/m^2^ and ≥ 2 metabolic risk factors. The MADO subjects were drug-naïve for anti-diabetic medications prior to recruitment. A written informed consent was obtained from the participants prior to their inclusion in the study. The study was carried out according to the Declaration of Helsinki and approved by the Institutional Ethics Committee (IEC). All subjects underwent clinical and biochemical assessment. Detailed anthropometry such as height, weight, BMI, waist circumference (WC), body fat percentage and blood pressure (mmHg) were recorded using the standard methods. The samples were collected after 10–12 h of overnight fasting. Study subjects were screened for glucose intolerance by standard oral glucose tolerance test (OGTT) using 75 g of anhydrous glucose as per the American Diabetes Association (ADA) criteria [17].

### Biochemical investigations

Various biochemical parameters such as fasting plasma insulin (FPI)  by electrochemiluminescence immunoassay and HbA_1C_ (Bio-Rad D10 system, Hercules, CA, USA) was determined by ion-exchange high-performance liquid chromatography (HPLC) method. Plasma glucose and lipid profile were estimated by an enzymatic method (Roche autoanalyser, Mannheim, Germany). The insulin resistance and β-cell function were calculated using the homeostasis model assessment HOMA-IR and HOMA-β by the standard formula [18].

### PBMCs isolation

Around 3–4 ml of whole blood was collected from all the study subjects. PBMCs were isolated using Ficoll–Hypaque (Sigma-Aldrich, St Louis, MO, USA) density gradient centrifugation method and were washed twice with 1X phosphate buffer saline (PBS) [19]. Cells were counted, and viability was assessed by trypan blue exclusion assay (> 95% PBMCs were viable). PBMCs obtained from the study subjects were used for determining the mitochondrial reactive oxygen species (mtROS) production and mitochondrial membrane potential (MMP), as well as mRNA and protein expression and TEM studies.

### Measurement of proinflammatory cytokines

Levels of IL-6 (BD Biosciences, USA) and highly sensitive C-reactive protein (hsCRP) (Diagnostics Biochem, Canada) were determined by sandwich enzyme-linked immunosorbent assay (ELISA) in MHNO, MHO and MADO subjects according to the manufacturer’s instructions. The samples were read on an ELISA microplate reader (TECAN, Salzburg, Austria) at a test wavelength of 450 nm.

### Mitochondrial reactive oxygen species (mtROS) measurement

Freshly isolated PBMCs were incubated with 5μM MitoSOX™ Red reagent (Molecular Probes, Invitrogen, CA, USA) for 10 min at 37 °C in a humidified 5% CO_2_ incubator, under dark conditions. After the completion of the incubation time, the cells were washed twice with 1X  PBS and subjected to flow cytometry (Becton Dickinson FACS Aria II) and the data was analyzed using BD FACSDiva software (Becton Dickinson, Franklin Lakes, NJ, USA).

### Detection of mitochondrial membrane potential (MMP)

Briefly for JC-1 staining, PBMCs were incubated with JC-1 stain (1:100 dilution in 1 × JC-1 assay buffer) (BD Biosciences, USA) for 10 min, suspended in 1 × JC-1 assay buffer at 37 °C with 5% CO_2_. The cells were washed twice with 1X  PBS and subsequently resuspended in 1 × assay buffer for flow cytometric analysis. The entire protocol was performed under dark conditions. Data were acquired using the BD FACS Aria II flow cytometer (Becton Dickinson Bio-sciences) and analyzed by BD FACSDiva software version 6.1.2 (Becton Dickinson, Franklin Lakes, NJ, USA).

### RNA isolation and real-time quantitative PCR (qRT-PCR)

Total cellular RNA was extracted from PBMCs by TRIZOL reagent (Life Technologies) and 2 µg of RNA was reverse transcribed to cDNA template by reverse-transcriptase PCR. Real-time PCR amplifications of mitophagy-related genes, i.e. *PINK1*, *PARKIN*, *NIX*, *MFN2*, *LC3*-*II* and *LAMP*-*2* were performed in duplicates in 20 µl reaction volume by SYBR green chemistry (Applied Biosystems). Human-specific primers were used (Applied Biosystems). The data is represented as relative mRNA expression normalized to human β-actin mRNA expression. The quantification of gene expression was calculated by 2^−ΔCT^ method, which is expressed in fold change [20]. The relative fold change in the MHO and MADO groups was normalized with the fold change of MHNO subjects.

### Western blotting

PBMCs were lysed in RIPA buffer (50 mM Tris–HCl pH 7.6, 150 mM NaCl, 1 mM EGTA, 1% Triton NP-40, 1% sodium deoxycholate, 0.1% SDS, 50 mM NaF), supplemented with protease inhibitors (Sigma-Aldrich, USA). Cell lysates were centrifuged at 10,000 rpm and the protein concentration was determined in the supernatant samples by Qubit reagent (Molecular Probes, Invitrogen, CA, USA). 70 µg of protein was resolved on 12.5% SDS-polyacrylamide gels and transferred onto PVDF membranes (Millipore, Bedford, MA) using a semi-dry transfer system (Amersham Biosciences, GE Healthcare, USA). PVDF membranes were blocked with 5% skimmed milk in Tris-buffered saline Tween buffer (TBST; 20 mM Tris, 137 mM NaCl, pH 7.6, with 0.1% TWEEN 20) overnight at 4 °C. The blots were incubated with primary antibodies against PINK1 (1 µg/ml; Sigma-Aldrich, USA), PARKIN (1 µg/ml), MFN2 (2.51 µg/ml), NIX (0.75 µg/ml), LC3-II (1.5 µg/ml) and LAMP-2 (2 µg/ml), obtained from Abcam (Cambridge, MA, USA), in TBST for 2 h at room temperature. After three washings with TBST, the membrane was probed with their respective HRP-conjugated secondary antibodies for 1 h at room temperature. Immunoblot images were detected using an ECL substrate (Thermo Scientific, Waltham, MA, USA) by an enhanced chemiluminescence system (Fluorchem M, Protein simple). The band intensity of target proteins was measured by Image J software (1.47 v) and normalized to levels of β-actin, which was used as a loading control.

### Transmission electron microscopy (TEM)

For TEM studies, the PBMCs collected from the study subjects were fixed with 3% glutaraldehyde in 0.2 M Sorensen’s phosphate buffer (pH 7.4) for 2–4 h, followed by 1% osmium tetroxide in Millionig’s buffer of the same pH for 2 h. The samples were subsequently dehydrated through an ascending ethanol series (70, 90, 100%), and embedded in an epon resin followed by polymerization at 60 °C for 24 h. Ultrathin sections (50 nm) were obtained using an ultramicrotome and mounted on the EM grids. The sections were stained with 1% uranyl acetate for 20 min and observed under a JEM 1400 Plus electron microscope (Jeol Ltd., Tokyo, Japan). The total mitochondrial number and percentage of damaged mitochondria per cell were evaluated by two independent examiners in a blinded manner. Image J software was used to assess the morphology of damaged mitochondria, which was defined by aspect ratio (length of the major axis/ minor axis) and form factor (perimeter^2^/4π * area), depicting the size and degree of mitochondrial branching, respectively.

### Statistical analysis

Data are expressed as mean ± SD for the parametric data and median (interquartile range) for the nonparametric variables. One-way analysis of variance (ANOVA) for parametric data, while Kruskal Wallis H test for non-parametric data was employed to compare the groups, followed by a post hoc test (Bonferroni or Dunn’s multiple comparison tests, respectively). Spearman correlation coefficient was calculated to see relationship between different variables. Values with p < 0.05 were considered statistically significant. The statistical analysis was performed using the SPSS version 22 software for window (SPSS Inc., Chicago, USA).

## Results

### Clinical and metabolic characteristics

The clinical and biochemical characteristics of the study subjects are summarized in Table 1. The anthropometric measurements including weight, BMI, WC, and body fat % were significantly higher in MHO and MADO subjects as compared to the MHNO group, while they were comparable between MHO and MADO subjects (p < 0.05). Further, other evidence for markers of insulin resistance like acanthosis nigricans and skin tags were observed to be highly prevalent in the MHO (50% and 15%) and MADO (85% and 45%) groups as compared to the MHNO subjects (30% and 5%), respectively. Systolic blood pressure (SBP) and diastolic blood pressure (SBP) were higher in the MHO and MADO groups, though non-significant. All subjects in MHNO and MHO group were normotensive, while 15% of MADO patients received anti-hypertensive drugs. The biochemical parameters including FPG, 2hPG and HbA_1C_ levels were significantly higher in the MADO group as compared to their MHO and MHNO counterparts (p < 0.05). The lipid and lipoprotein profile was comparable between the MHNO and MHO subjects, while the MADO subjects displayed higher levels, though non-significant, except for LDL-C levels, which were significantly higher as compared to the other groups (p < 0.05). Interestingly, the MHO subjects displayed a preserved insulin sensitivity as reflected by the comparable HOMA-IR indices relative to the MHNO participants. However, the MADO subjects displayed higher levels of HOMA-IR than the other groups (p < 0.05). Further, HOMA-β indices were significantly reduced in MADO group as compared to the MHO and MHNO subjects (p < 0.05).

**Table 1:** Clinical and biochemical characteristics of metabolically healthy non-obese (MHNO), metabolically healthy obese (MHO) and metabolically abnormal diabetic obese (MADO) subjects

Variables	MHNO (n = 20)	MHO (n = 20)	MADO (n = 20)
Age (years)	38.9 ± 7.2	41.5 ± 6.5	43.7 ± 10.8*
Gender (M:F)	12:8	11:9	11: 9
Wt (kg)	63.8 ± 7.7	72.2 ± 8.3*	73.1 ± 10.5*
BMI (kg/m^2^)	21.2 ± 1.6	28.5 ± 2.4***	29.4 ± 3.5***
WC (cm)	81.0 ± 5.2	96.3 ± 7.7***	98.6 ± 8.0***
Body Fat (%)	22.8 ± 6.2	33.6 ± 7.4*	35.0 ± 10.4***
Systolic BP (mm/Hg)	119.7 ± 12.1	122.7 ± 10.7	126.2 ± 15.2
Diastolic BP (mm/Hg)	80.7 ± 7.9	85.0 ± 6.1	81.8 ± 9.6
FPG (mmol/l)	5.1 ± 0.4	5.2 ± 0.7	8.6 ± 1.4***^###^
2hPG (mmol/l)	5.6 ± 1.3	6.8 ± 1.2	13.6 ± 2.7***^###^
HbA_1C_ (%)	5.2 ± 0.2	5.3 ± 0.4	7.7 ± 0.5***^###^
FPI (μIU/ml)	9.7 ± 6.2	11.9 ± 5.1	13.6 ± 4.7
HOMA-IR	2.2 ± 1.4	2.4 ± 1.0	5.4 ± 2.5***^###^
HOMA-β %	119.5 ± 83.6	156.3 ± 81.1	54.2 ± 17.6***^###^
S. CHOL (mg/dl)	185.2 ± 39.1	180.4 ± 37.5	206.9 ± 44.8
S. LDL-C (mg/dl)	112.5 ± 27.9	108.1 ± 32.2	141.0 ± 36.7*^##^
S. TG (mg/dl)	134.2 ± 77.8	140.4 ± 70.2	152.2 ± 57.3
S. HDL-C (mg/dl)	44.2 ± 7.7	50.1 ± 16.7	44.2 ± 6.7
IL-6 (pg/ml)	6.9 ± 1.1	8.2 ± 2.2	15.2 ± 2.6**^#^
hsCRP (mg/dl)	0.7 ± 0.4	1.7 ± 1.2*	3.8 ± 2.3***^#^

Data expressed as mean ± SD, * p < 0.05, ** p < 0.01, *** p < 0.001 vs. MHNO

^#^p < 0.05, ^##^ p < 0.01, ^###^ p < 0.001 vs. MHO

### Levels of proinflammatory cytokines

MHO and MADO groups exhibited significantly higher serum levels of hsCRP relative to the MHNO subjects (p < 0.05). IL-6 levels were higher, though insignificant in MHO subjects as compared to the MHNO group, while they were significantly higher in the MADO group as compared to the other groups (p < 0.05) (Table 1).

### mtROS content and MMP

**Fig. 1:**
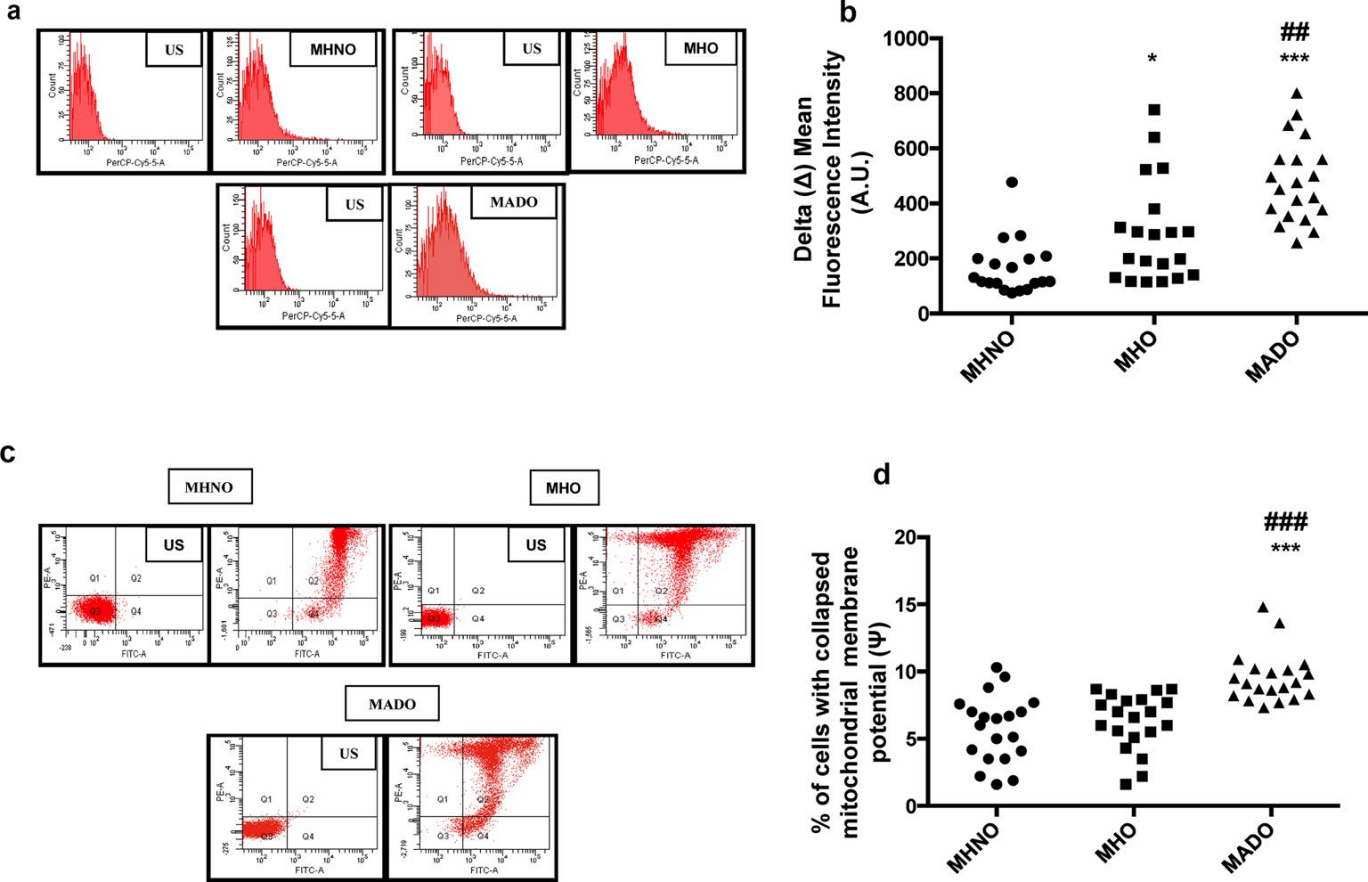
Depicts mtROS content and MMP in the study subjects. **a** Representative FACS image of Mitosox fluorescence intensity. **b** Bar graph represents delta (Δ) mean fluorescence intensity of Mitosox in metabolically healthy non-obese (MHNO), metabolically healthy obese (MHO) participants and (n = 20 each) and metabolically abnormal diabetic obese (MADO) participants. **c** Representative scatter plot of MMP. **d** Bar diagram showing the percentage of cells with collapsed MMP in MHNO, MHO and MADO subjects. Values are expressed in median and interquartile range (n = 20 each), (* = vs. MHNO), (^#^ = vs. MHO), *p < 0.05, ***p < 0.001, ^##^p < 0.01, ^###^p < 0.001

As compared to the MHNO group, the MHO subjects exhibited higher mitochondrial oxidative stress levels as evidenced by significantly increased mtROS production (p < 0.05). However, MMP, which is expressed as the percentage of cells with collapsed membrane potential, showed a moderate increase in the MHO subjects, though it was insignificant. Further, the mtROS content and collapsed MMP were significantly higher the in MADO subjects (p < 0.05 vs. both MHNO and MHO groups) (Fig. 1a–d).

### Correlation between body composition, HbA_1C_ and mitochondrial ROS content

**Fig. 2:**
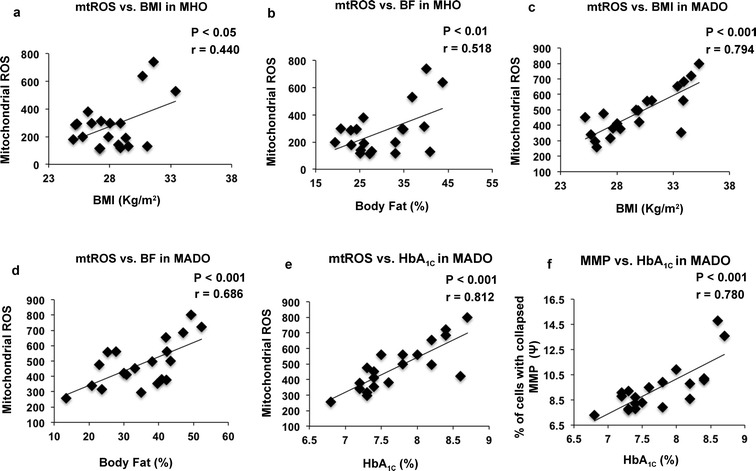
Scatter plot illustrating the correlation of mitochondrial ROS (mtROS) vs. BMI and body fat in the MHO group (**a**, **b**), BMI and body fat in the MADO group (**c**, **d**). Correlation graph displaying the relationship between the increasing HbA_1C_ levels and mtROS content, and MMP in the MADO subjects (**e**, **f**)

A significant and positive correlation of BMI (r = 0.440; p < 0.05) and body fat percentage (r = 0.518; p < 0.01) with the increased mitochondrial ROS content was observed in the MHO participants. Similar correlation was also noted with BMI (r = 0.794; p < 0.001) and body fat percentage (r = 0.686; p < 0.001) in the MADO patients. Though the severity of mitochondrial oxidative stress was more, as MOS also showed a positive correlation with the rising HbA_1C_ levels, reflected by MMP (r = 0.780; p < 0.01) and mtROS (r = 0.812; p < 0.01) in these patients (Fig. 2a–f).

### Transcriptional profiling of mitophagy-related genes

**Fig. 3:**
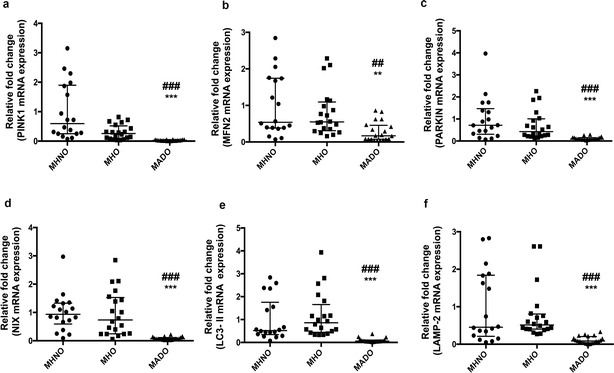
Depicts mRNA expression of mitophagy markers **a** PINK1, **b** MFN2, **c** PARKIN, **d** NIX, **e** LC3-II and **f** LAMP-2 in MHNO, MHO and MADO subjects. Values are expressed in median and interquartile range, (n = 20 each), (* = vs. MHNO), (^#^ = vs. MHO), **p < 0.01,***p < 0.001, ^##^p < 0.01, ^###^p < 0.001. The relative mRNA levels in MHO and MADO subjects has been normalised with the gene expression of MHNO subjects

mRNA expression of PINK1 gene was significantly attenuated in the MHO subjects as compared to the MHNO group (p < 0.05). However, no significant alterations in the mRNA expression of other mitophagy-related markers including MFN2, PARKIN, NIX, LC3-II and LAMP-2 genes was observed between both the MHO and MHNO subjects, whereas, a significant downregulation in the mRNA levels of mitophagy-related genes was observed in the MADO patients with respect to MHNO and MHO groups (p < 0.05) (Fig. 3a–f).

### Translational profiling of mitophagy-markers

**Fig. 4:**
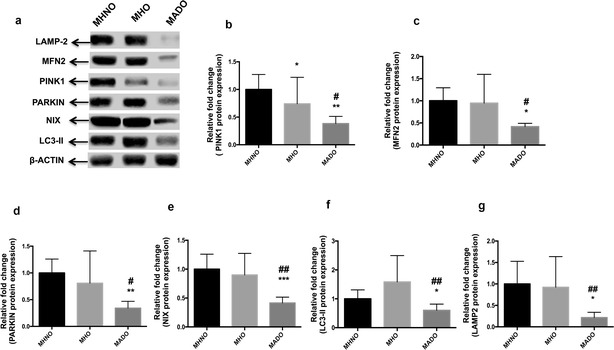
**a** Representative blots of mitophagy markers in MHNO, MHO and MADO subjects (n = 10 each). Bar graphs showing protein expression of **b** PINK1, **c** MFN2, **d** PARKIN, **e** NIX, **f** LC3-II and **g** LAMP-2 in MHNO, MHO and MADO subjects. Values are expressed in mean ± SD, (n = 10 each), (* = vs. MHNO), (^#^ = vs. MHO), *p < 0.05, **p < 0.01, ***p < 0.001, ^**#**^p < 0.05, ^##^p < 0.01

Western blot analysis revealed that there was no significant difference in the protein expression of PARKIN, MFN2, NIX, LC3-II and LAMP-2 except for PINK1 protein, which was significantly downregulated in the MHO subjects as compared to the MHNO group (p < 0.05). Moreover, MADO group displayed a significantly attenuated expression profile of mitophagy-related proteins as compared to the other groups (p < 0.05) (Fig. 4a–g).

### Quantification of damaged mitochondria

**Fig. 5:**
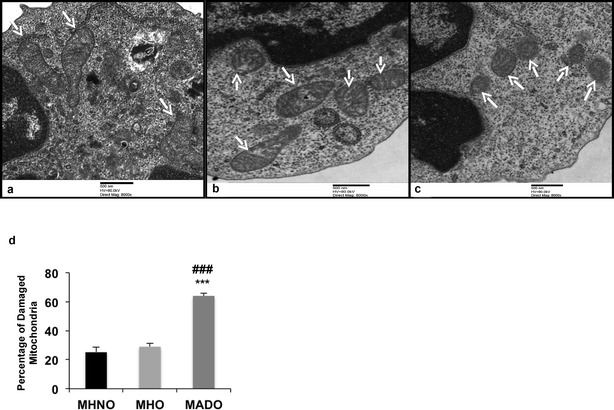
Electron micrographs of PBMCs showing mitochondria in **a** MHNO, **b** MHO and **c** MADO groups. Mitochondria are represented by white arrows. Magnification: ×8000, Scale bar: 500 nm. MHNO and MHO groups displayed healthy mitochondria, which are larger in size, elliptical/oval in shape with well-preserved cristae, while MADO patients showed smaller and fragmented mitochondria. **d** Bar diagram displaying the percentage of damaged mitochondria in the respective study groups. Data expressed as mean ± SD  (n = 3 each), (* = vs. MHNO), (^#^ = vs. MHO), ***p < 0.001, ^###^p < 0.01)

TEM analysis demonstrated that there was no significant difference in the total mitochondrial number per cell among all the study subjects. However, a significant increase in the percentage of damaged mitochondria was observed in the MADO patients relative to the MHO and MHNO groups, as depicted by significantly reduced aspect ratio and form factor (p < 0.05). However, subjects in MHO and MHNO groups displayed almost similar percentage of damaged mitochondria (Fig. 5a–d; Table 2).

**Table 2:** Mitochondrial morphological characteristics in metabolically MHNO, MHO and MADO subjects

Morphological characteristics	MHNO (n = 3)	MHO (n = 3)	MADO (n = 3)
Mitochondria (#/cell)	6.8 ± 1.5	7.2 ± 1.3	7.1 ± 0.5
Aspect ratio	1.81 ± 0.5	1.84 ± 0.9	1.20 ± 0.4**^#^
Form factor	1.63 ± 0.1	1.62 ± 0.5	1.16 ± 0.4**^##^
% of damaged mitochondria	25.2 ± 3.3	29.1 ± 2.3	64.0 ± 1.2***^###^

Data expressed as mean ± SD, ** p < 0.01, *** p < 0.001 vs. MHNO

^#^p < 0.05, ^##^ p < 0.01, ^###^ p < 0.001 vs. MHO

### Correlation between the damaged mitochondria and mitophagy

Correlation analysis revealed that the percentage of damaged mitochondria showed a significant and negative correlation with the mRNA levels of mitophagy-related markers including NIX (r = − 0.702; p = 0.035), PARKIN (r = − 0.680; p = 0.044), LC3-II (r = − 0.761, p = 0.017) and LAMP-2 genes (r = − 0.707; p = 0.033).

## Discussion

The present study demonstrates that obesity is associated with an increased mitochondrial oxidative stress, but did not significantly influence mitophagy in MHO subjects. However, the MADO patients with similar body composition as MHO group were accompanied by metabolic abnormalities, increased levels of mitochondrial oxidative stress, and displayed attenuated mitophagy. Our observations indicate that obesity-mediated oxidative stress doesn’t significantly alter mitophagy, unless accompanied by metabolic abberations as seen in the MADO patients. Therefore, it is conceivable that the favorable metabolic profile contributes to sustenance of mitophagy in response to mild oxidative stress levels in the MHO group and this may further limit the rising mitochondrial oxidative stress in these subjects.

Obesity-mediated oxidative stress has been linked to metabolic diseases like diabetes and atherosclerosis, as increased adiposity in the obese individuals results in an increased oxidative stress and mitochondrial dysfunction, an underlying cause of insulin resistance [21, 22]. Our data revealed that the MHO subjects exhibited increased mitochondrial ROS content, which showed a positive and significant correlation with BMI and body fat percentage. However, a non-significant increase in the collapsed MMP in MHO subjects could be attributed to the sustained mitophagic process, resulting in the engulfment of the damaged and defunct mitochondria. This phenomenon may result in limiting the aggravated mitochondrial oxidative stress levels, as these defunct mitochondria are the potential source of ROS generation. These observations indicate that the MHO subjects display a higher degree of mitochondrial oxidative stress as compared to the MHNO subjects due to the higher BMI and body fat percentage. However, MHO subjects have been shown to display moderately lower levels of oxidative stress as compared to the MADO individuals, with similar BMI and body fat, though accompanied with altered metabolic profile. In this context, Bañuls et al. also reported that the mitochondrial ROS production was enhanced in MADO subjects as compared to the MHO group [23]. Our study revealed that MADO patients exhibited even higher mtROS content and collapsed MMP as compared to the MHNO and MHO groups. Further, mitochondrial stress indices also showed a positive correlation with body composition and rising HbA_1c_ levels, indicating that the adiposity accompanied with glucotoxicity may have contributed to an aggravated mitochondrial oxidative stress in these patients.

Reports in the literature demonstrate that the MHO individuals may not be predisposed to the similar risk of developing metabolic abnormalities as compared to the MADO subjects. Nonetheless, the underlying mechanisms for the apparently healthy metabolic profile of the MHO subjects remain conjectural. Accumulating evidence reports that the preferential autophagic processes like mitophagy are triggered in response to ROS [24]. To the best of our knowledge, this is the first report in the literature to assess the status of mitophagy in metabolically healthy obese (MHO) subjects, particularly in Asian Indians. Our observations revealed that the mRNA and protein expression of mitophagy markers in MHO subjects remained unaltered and akin to that of the MHNO subjects, except for PINK1, which was significantly downregulated in MHO group. However, in patients with MADO, the expression of mitophagy-related markers was significantly attenuated as compared to MHO and MHNO groups. Sustenance of mitophagy in MHO participants can be explained by the fact that the magnitude of mitochondrial oxidative stress was moderate, and may not have attained a particular threshold level that could have significantly altered the mitophagy. Moreover, the favourable metabolic phenotype in these MHO subjects may also have contributed to the the sustenance of mitophagy by limiting ROS generation. Nevertheless, attenuated PINK1 expression in MHO subjects may be explained by the fact that PINK1 is highly susceptible to oxidative stress-induced proteolysis, thereby, rendering it non-functional [25]. Interestingly, the HOMA-IR levels were not significantly different in MHO subjects as compared to the MHNO subjects, indicating a crucial role of mitophagy in maintaining mitochondrial health and consequent insulin sensitivity. As far as MADO patients are concerned, multiple metabolic abnormalities and higher levels of mitochondrial oxidative stress may result in oxidative damage of mitochondrial proteins, which generates “mitophagy-resistant” mitochondria, leading to impaired mitophagy and consequent accumulation of dysfunctional mitochondria and increased ROS generation. Our findings indicate that phenotypic alterations are not suffice to exert significant impact on mitophagy unless accompanied by severe metabolic abnormalities as observed in patients with MADO.

Further, TEM analysis demonstrated an increased number of damaged mitochondria in patients with MADO and also showed a negative correlation with the mitophagy-related markers, indicating that metabolic abnormalities-induced mitochondrial oxidative stress led to attenuated mitophagy, resulting in the accumulation of dysfunctional mitochondria. Of note, the alterations in mitochondrial oxidative stress and mitophagy are of particular importance in PBMCs of MHO and MADO subjects, as these cells have been shown to reflect oxidative stress and inflammation associated with obesity [26, 27].

Obesity is also a state of chronic inflammation and contributes as a major risk factor for the development of T2DM. Therefore, there is a likelihood that inflammasome regulation plays an important role in the induction and pathogenesis of the inflammatory disorders like obesity and diabetes [28]. Moreover, a recent report by Kim et al. revealed that mitophagy negatively regulates inflammasome activation by engulfing the damaged mitochondria, as these superfluous mitochondria act as potential activators of inflammasomes [29]. Though we did not assess the inflammasome activity in the study subjects; our data demonstrated that sustained mitophagy in MHO subjects may inhibit inflammasome activation, which may further reduce inflammation in these subjects, despite having higher BMI. Thus, our observations indicate that mitophagy, a cell-reparative process, is a self-limiting phenomenon that may protect the cells from excessive inflammation, contributing to a low-grade inflammatory profile in MHO subjects.

In conclusion, our study highlights the importance of favourable metabolic profile in MHO subjects, which may be one of the contributing factor in the sustenance of mitophagic process, a beneficial metabolic event. This phenomenon results in reduced mitochondrial oxidative stress, low-grade inflammation, thereby, leading to improved mitochondrial function and preserved insulin sensitivity. Thus, these findings strengthen the understanding of pathophysiological mechanisms underlying the reduced risk of developing metabolic abnormalities due to lower oxidative stress and inflammatory profile in the MHO subjects.

## Abbreviations

MHNO: metabolically healthy non-obese
MHO: metabolically healthy obese
MADO: metabolically abnormal diabetic obese
T2DM: type 2 diabetes mellitus
ROS: reactive oxygen species
HOMA-IR: homeostatic model assessment of insulin resistance
HOMA-β: homeostatic model assessment of β-cell function
mtROS: mitochondrial reactive oxygen species
MMP: mitochondrial membrane potential
